# Energy Management and Control in Multiple Storage Energy Units (Battery–Supercapacitor) of Fuel Cell Electric Vehicles

**DOI:** 10.3390/ma15248932

**Published:** 2022-12-14

**Authors:** Khairy Sayed, Sayed Abdel-Khalek, Hesham M. H. Zakaly, Mahmoud Aref

**Affiliations:** 1 Electrical Engineering Department, College of Engineering, Sohag University, Sohag 1646130, Egypt; 2 Department of Mathematics, College of Science, Taif University, P.O. Box 11099, Taif 21944, Saudi Arabia; 3 Physics Department, Faculty of Science, Al-Azhar University, Assiut Branch, Assiut 71524, Egypt; 4 Department of Electrical Engineering, Assiut University, Assiut 71516, Egypt; 5 Department of Automated Electrical Systems, Ural Power Engineering Institute, Ural Federal University, 620075 Yekaterinburg, Russia

**Keywords:** electric vehicle, automotive applications, DC power supply, supercapacitor, efficiency, energy management system

## Abstract

This paper presents a new approach of energy management for a fuel cell electric vehicle traction system. This system includes a supercapacitor, a traction battery of valve-regulated sealed lead–acid type, a high-performance permanent magnet traction system, and a power electronics converter. Special attention was placed on the coordination for managing the flow of energy from several sources to treat the concerns of prolonged electric vehicle mileage and battery lifetime for drivetrains of electric vehicles. Connection to a supercapacitor in parallel with the electric vehicle’s battery affects electric vehicle battery lifetime and its range. The paper used a study case of an all-electric train, but the used methods can be applied on hybrid or electric train cases. Fuzzy logic control and proportional integral control methods were used to control the electric vehicle system. The results of these two control methods were examined and compared. The simulation results were compared between the proposed electric vehicle system and the traditional system to show the effectiveness of the proposed method. Comparison of waveforms was made with and without the supercapacitor. The proposed optimized energy management strategy could improve the overall performance of the hybrid system and reduce the power consumption.

## 1. Introduction

The widespread use of fuel cells in power generation and reprocessing is because of their short-term refueling, hydrogen refueling, minimal noise pollution during operation, water-only production, and operation with a wide range of environmentally friendly hydrogen sources. Traditionally, the energy storage requirements of conventional and fuel cell hybrid buses have been covered by chemical cells. This is because, among other things, of low acquisition costs, high energy densities, and a high level of engineer knowledge compared to new technologies. However, batteries have many drawbacks, such as restricted lifetime, maintenance, conditioning constraints, and moderate power density, which are improved by new technologies such as supercapacitors and high-speed flywheels [1,2]. Supercapacitors may be utilized as a momentary power source for backup solutions instead of batteries in some specialized applications. However, the excellent energy storage capacity of supercapacitors makes them a good alternative to batteries. In these applications, supercapacitors are charged from the grid to provide temporary power in the event of a power outage [3]. The energy storage system of hybrid electric vehicles is second application of the ultra-capacitor. Supercapacitors are used to deliver a short burst of energy required by a hybrid electric vehicle in speeding up. Supercapacitors are less affected by repeated deep charging and discharging, and therefore they do not need to be replaced on a regular basis like batteries. This also indicates that supercapacitors are more ecologically friendly as they do not need to be disposed of regularly. Supercapacitors can be completely charged within seconds of deep discharge. For that, they are perfectly used in regenerative braking applications. Ultra-capacitors cannot completely replace batteries. However, they can be employed to match together. In hybrid electric vehicles, supercapacitors can be used while driving the vehicle. When the vehicle is stopped, a smaller battery can be used to supply the auxiliary system. The slow time of charge can be used to recharge the battery and extend its life. Batteries cannot conduct large currents at extremely low temperatures, but they can carry enough current to slow charging supercapacitors that can start the electric vehicle. This can make it easier to use batteries with a long service life, rather than large batteries that are designed for hundreds of amperes at cold start.

In recent years, there have been some successful demonstrations of FC-based marine applications [4,5,6,7]. To date, most departing FC vessels have been powered by cold FC for zero emissions, relatively high-power densities, and high-speed takeoff. The pure hydrogen required for this technology can be stored on a ship relatively easily, making it particularly suitable for low-power ships, such as small sports ships and passenger ships. On the other hand, high temperature FC power systems such as MCFCs, SOFCs, and PEMFCs with reformers are suitable for ships in large power plants. In this case, these FCs can be supplied with conventional fuels such as diesel and gas. Some of the feasibility, assessment, and design implications are described in [8,9,10,11]. The FC energy system has an excellent ability to track energy demand under steady-state operation conditions, but its dynamic response to transient energy demand is relatively weak. Dynamic fluctuations in power requirements place a heavy load on the FC membrane and shorten the useful life of the FC power supply [3]. In addition, FCs used in ships are more costly than traditional internal combustion engines and cannot meet current peak performance requirements. This means that the FCs can be connected as a hybrid to the battery bank to cover the entire energy requirements. The hybridization degree is the ratio of FC energy to the total energy requirement of a hybrid electric vehicle (HEV) and can be determined depending on the role of the energy storage system. FCs typically need to provide minimal continuous energy, and energy storage systems cover peak or accelerated power demand. For marine applications, the primary drive for FC must supply at least mobile power. Otherwise, this leads to a significant increase in the energy consumption of energy storage systems. Boats, on the other hand, have more energy storage space than HEVs, so they can carry larger batteries.

To meet the requirements of [12], a hybrid FC battery power system with a single DC/DC boost converter was presented. Energy management systems (EMS) based on fuzzy logic are used in FC/battery/supercapacitor hybrid buses [13] to distribute power to various power sources. A comparison of various HEV energy management strategies is shown in [14]. This paper focuses on using hybrid propulsion configurations for low power boats. The purpose of this paper was to investigate the performance of such hybrid systems that connect FCs to batteries to identify the propulsion of low power recreation boats. This case study proposes an efficient optimal EMS based on operating conditions. This EMS technique defines the operating point of each component in the system to maximize the efficiency of the system.

Apart from the circuit topology of hybrid power supply systems, the latest control strategies used to control ultracapacitor/battery systems and model predictive control (MPC) systems for hybrid batteries/ultracapacitor power supplies [15] were proposed. The contribution of the MPC process is that the state of charge of the battery and the ultracapacitor current and ultracapacitor voltage are kept within predefined limits during operation. In addition, the controller has the ability to instantly charge and discharge current, assigning rapid current changes to supercapacitors. From the previous studies above, it may be found that much of the study is conducted in the context of hybrid energy storage systems (HESS) [16,17,18,19]. However, the distribution of brake energy between the UC and the battery is an important issue, especially at regenerative braking mode EV. Due to the variation of HESS parameters, it is also very difficult to obtain a precise model of HESS, and unmodeled components require a more robust controller. The ripple current that flows through the battery during charging mode can produce heat (I2R loss) by interacting with the battery’s internal resistance. This heat contributes to the internal heat generated by the battery. Therefore, if the ripple current is too high, the battery life will be shortened [20]. Battery manufacturers recommend that the charging voltage of the battery should be under normal float charging conditions. It is necessary to limit the DC-voltage ripple applied to the battery to about 0.5% [21]. This guarantees that the current cell voltage does not decrease below the open-cell voltage or exceed the maximum voltage of float charge. It also reduces the subsequent battery overheating caused by the constant circulation of the battery between the discharged and charged states. Ripple current has many drawbacks. The increased ripple current through the circuit components reduces the useful life of the converter. Battery life is limited by the discharge/charge effect. In addition, ripple during charging reduces charging performance. The same ripple occurs on the connected load.

The power electronics subsystem of an electric vehicle (EV) powertrain must control both the flow of energy in the vehicle and the transmission of torque by electrical machinery [22]. Such systems are known to produce unwanted electrical noise on high-voltage buses. The vibration or ripple of the high frequency current enters the vehicle’s battery system unimpeded. Actual measurements of the high-voltage bus currents of series hybrid electric vehicles (HEVs) show that large current failures occur in the range from 10 Hz to above 10 kHz. Little has been reported in the scientific literature on the potential impact on the performance of battery system and degradation rates related with battery exposure to combined direct current (DC) and alternating current (AC) currents. A key technology in this area is the design and integration of the energy flow within the vehicle and the power electronics subsystem required to control the generation of torque by the electromechanical during vehicle acceleration and regenerative braking. A comprehensive overview of the various hybrid powertrain architectures for commercial vehicles and a detailed description of power electronics components can be found in [23].

Such electric powertrains typically include advanced electronics and power electronics components such as insulated gate bipolar transistors (IGBTs) and field effect transistors (FETs), bidirectional DC for electromechanical machines. It is integrated into both DC converters and inverter drives [24]. Orders of 20 kHz up to 50 kHz work within the vehicle application [25]. This switching operation is known to induce high-frequency harmonic signals coupled to DC battery current, coupled with a highly reactive load connected to the vehicle’s electrical machinery [26]. Rechargeable batteries used in both electric vehicles and HEVs are often characterized by a useful life defined by the number of continuous charge/discharge cycles for a given capacity loss [27,28]. Therefore, this task will carry out a new investigation into the long-term performance degradation of lithium-ion battery cells when exposed to coupled AC–DC current waveforms that represent actual HEV usage. This task quantifies battery degradation caused by the coupling of AC–DC excitations resulting from current measurements on the HEV’s high-voltage bus in a temperature-controlled system.

This article describes the way in which to add a supercapacitor to the powertrain of a fuel cell vehicle. We compared the waveforms with and without supercapacitors. Using a supercapacitor pack in parallel with an electric vehicle battery pack can affect battery life and vehicle range. A new battery supercapacitor system was proposed with a new algorithm that determines when to charge and discharge the battery or supercapacitor according to operating conditions such as acceleration and deceleration to extend the range of the modified vehicle. The simulation was performed to demonstrate drive cycle. The performance of the proposed algorithm was asserted by comparing the simulation results of the studied system with the conventional battery–electric vehicle system. This new system can reduce the load on the battery and extend the cruising range of the vehicle compared to traditional battery-powered electric vehicles.

## 2. Materials and Methods

### 2.1. Fuel Cell Vehicle Drive-Train Configurations

Regenerative braking is an important performance issue for all-electric vehicles, as energy savings directly correlate with the vehicle’s range. Generally, the highly dynamic performance requirements inherent in urban driving can have a substantial influence on battery energy usage, especially for lead–acid battery technology. In particular, high-pulsed power transients from lead–acid batteries can limit the full use of the energy stored in the battery, even for short periods of time (2–3 s) [6].

**Figure 1:**
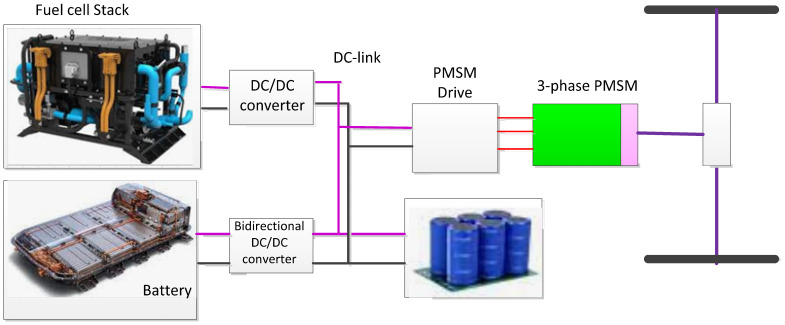
Configurations of a fuel-cell-powered electric vehicle.

Figure 1 shows the most frequently selected powertrain connection scheme, especially for all electric vehicle formats. It shows two options for connecting onboard energy and power storage choices participating in a “series” powertrain configuration. For completeness, the location of the internal combustion engine or fuel cell in the drivetrain for the hybrid is also shown. The energy management attitude is that the internal combustion engine or fuel cell performs a function like an electrochemical storage battery by providing the average energy demand of the vehicle, and the complete peak power buffer delivers a transient energy supply acceleration or regenerative braking. This regards the connection diagram of the figure. In Figure 1, the energy storage device provides the intermediate circuit voltage directly to the power electronics inverter of the traction machine, and the peak power buffer option is connected to a DC–DC power converter. In this structure, the power supply voltage is fairly well limited, the voltage drop from full charge to full discharge of the traction battery under consideration is typically about 20%, and the fuel cell and ICE/generator optional voltages have more severe regulations to ensure that they work within the optimal efficiency window. In addition, the maximum power buffer voltage varies to near zero, maximizing the energy reserved from the buffer. However, the main drawback of this structure is that the maximum power buffer passes through a DC–DC converter designed for powertrain peaks (that is, it passes through a DC–DC converter).

Since the terminal voltage of the traction battery fluctuates greatly between the states of being completely charged and discharged, the traction drive must be designed to accommodate a wide operating voltage range without losing power. Therefore, switching from the traditional approach to a continuously changing DC-link approach, as shown in Figure 1, minimizes the influence on the design of the traction drive. In addition, the directly connection the peak power supply and the traction system is high energy performance as it significantly reduces the power requirements of the DC–DC converter, ideally only the average power of electric vehicle from the traction battery, NS. About 45 kW is transmitted. As a result, DC–DC converters can reduce 10 kVA of silicon power, and this is an important commercial aspect [2,6].

A permanent magnet synchronous motor (PMSM) is employed to propel electric vehicles powered by a proton exchange membrane (PEM) fuel cell. The PMSM has remarkable features, including high efficiency, high torque per unit volume, high power density, small size, and light weight, which meet the requirements of electric vehicles. Hence, it is recognized as a worthy choice for EVs and is widely used in new fuel cell vehicles.

To address the technical challenges of powertrain component integration and energy management method research, while providing validation of electric vehicle simulation tools, this system consists of two series-connected supercapacitor banks: a 135 V bank of 2500 F 50x Maxwell cell and 135 V bank of 350 F 300x SAFT cell connected in series to supply DC power to the traction system. The supercapacitor bank is connected to the individual DC–DC converters in the 2x Hawker sealed lead–acid battery pack (to accommodate variations in supercapacitor specifications). DC-link offers brushless permanent magnet traction machines suitable for vehicles and inverters. Its mechanical performance is loaded by the test bench test system. The specification of the studied system is listed in Table 1.

### 2.2. Control Strategy for Fuel Cell Drive-Train

**Figure 2:**
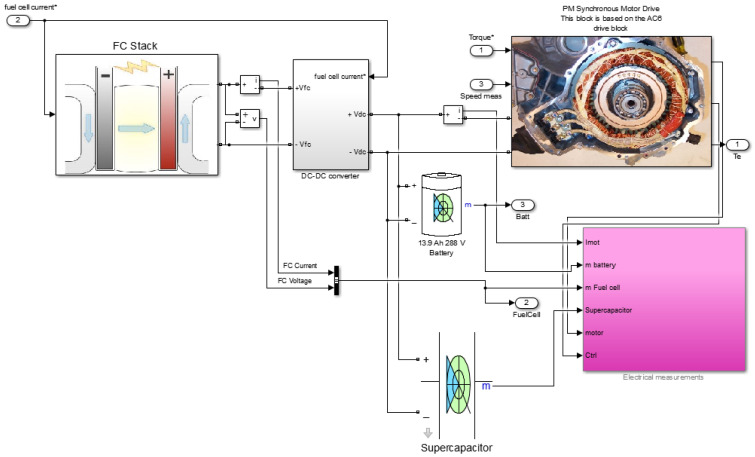
Power flow simulation model of an electric vehicle drive−train.

Originally, the experimental drive-train energy management scheme was a comparatively simple voltage tracking regulator with a low-pass filter applied to the dynamic drive power demand. This scheme allows for functional testing of combined drive-train elements but is not enhanced from an energy management perspective. The results show how expected battery power reductions and simple energy management strategies use battery power as well as regenerated power to charge supercapacitors. However, experiments with different drive cycles have shown that this simple energy management does not use all the existing power buffer rate. The DC-link voltage is kept around a predefined set value, but deviations above the set value are negative, whether this is the result of short-term regenerative braking or gain of long-term net energy. It is canceled by the control action of disconnecting energy from the maximum performance buffer. To examine the proposed control scheme performance, a simulation model of an electric vehicle with a fuel cell battery is shown in Figure 2.

The system control requirement is to keep the peak power buffer output voltage within the required limits while ensuring unipolar battery current, NS. There is no battery regeneration, and battery current is kept to a minimum. Driving cycles that are assumed to be unknown to vehicle control represent system performance failures. This scheme is particularly attractive for this application area, and thus a variant of MPC [28] with zone control is used. The NiMH battery has high energy density and abuse resistance but has a high discharge rate. Polymer lithium-ion batteries have up to four times the power density of lead batteries [29]. As a result, lithium-ion batteries are presently the maximum feasible choice for storing electricity in maximum business electric-powered vehicles. Table 1 suggests the traits of numerous electricity garage devices. Supercapacitors are excessive electricity density electricity garage structures that have obtained the eye of many current studies. Supercapacitors (SCs) are in many approaches, much like batteries. The major distinction is that, in contrast to batteries, supercapacitors cannot shop large quantities of electricity over a protracted length of time; because of this, they have a low electricity density [30]. The battery has a better electricity density and a medium electricity density. These residences have the cap potential to offer the car enough variety in line with complete charge (in terms of the use of a battery %, this is appreciably lighter than lead–acid batteries) and slight acceleration. However, the slight output density of lithium-ion batteries limits the car with excessive acceleration and excessive regenerative braking efficiency. Most of the regenerated braking pressure is misplaced at the wheel because of friction and heat, which the lithium-ion battery cannot do.

With the above facts in mind, this study proposes a control algorithm to extend the life of the EV battery and extend the cruising range of the EV. This is achieved by sudden decreasing surges of current up and down that are pulled out and perceived by the battery when the battery accelerates and slows down during regenerative braking. To achieve this, a supercapacitor (SC) package was combined with an EV battery. SCs are widely used in industrial applications because of their high pulse current, mutual discharge capacity, high cycle capacity, low equivalent series resistance (ESR), and recyclability. Using the SC pack with the battery also reduces the heat generated by the I2R loss in the HESS [31]. However, the main goal of using SC remains the buffering energy between the battery pack and the motor drive because of its low energy density [32]. The control algorithm determines which source is selected to obtain the required energy during the acceleration of the electric vehicle and which source is selected to provide extra energy during the deceleration and regeneration of the electric vehicle. Therefore, the control method is designed to take full advantage of the SC package. SC packs have a longer lifespan than lithium batteries [33]. Therefore, control algorithms allow the SC-Pack to consume sudden extra energy and direct this energy to future energy needs.

### 2.3. Optimal Control and Energy Management

In the AC microgrid system, the key to its operation is intelligent control and management [34,35,36,37,38,39,40,41]. The purpose of the controller is to tune distributed microgrid terminals to reduce power outages and uncertainties. Many AC microgrid control strategies can be used to keep a stable, reliable, and economical energy source for both local customers and utilities. However, because of the special characteristics of the DC system described above, it is not easy to migrate the control strategy of the AC microgrid to the DC microgrid. Similar to the AC grid, the control methods for DC microgrid systems can be divided into two classes of control structures: centralized and distributed. For centralized control, all micro source converters that share as DC bus are controlled by a central power controller via a communication link (such as the CAN bus). A server is required to collect information on each converter about its voltage, current, and node addresses. This solution suffers from power converter node limitations, node expansion difficulties, and the requirement for extra central control and hardware circuitry for high-speed communication. In comparison with centralized solutions, a distributed control structure was proposed in which all distributed terminals work autonomously, and control solutions depend on local data [18]. The control techniques are used in DC microgrids for energy management to control droops, voltage/current (V/I) and voltage/power (V/P). The central problem with U/I and U/P control techniques is the use of DC bus voltage deviations for independent distribution between various energy sources [42]. The restriction of the V/I and V/P structures is that all terminals using the same DC bus have a hypothetical droop curve without an elastic mode transmission mechanism; especially when voltage changes occur in the DC microgrid, this is necessary to strictly follow [20]. From [5], the proposed operating modes of the system when the DC-link voltage is tuned by various power supplies are as follows: (B) dominant mode of the energy storage system; (C) dominant mode of battery test equipment. Therefore, it is important to seamlessly transition from one dominant mode to another, depending on the voltage deviation of the DC bus. Performance variables for different terminals can be defined as follows:

PESS_CH and PESS_DSC—The maximum charging and discharging power values. These values are determined by the state of charge (SOC) of the energy storage system. A zero SOC for the battery and ultracapacitor means that the ESS is completely discharged, PESS_DSC = 0.

Vdcbus—DC-bus voltage; Pload-Power is the consumption of local loads, including linear and non-linear loads that share the same DC bus. Vupper and Vlower—Operation mode threshold DC-link voltage.

PBTS_CHG and PBTS_DSC—Total charge and discharge power. Table 2 shows the characteristics of each dominant mode determined by the DC-link voltage range. This shows that the performance characteristics of each terminal determine the rise and fall of the DC-link voltage.

In Mode I, when the intermediate circuit voltage falls below the lower limit, the utility dominates the intermediate circuit voltage. In Mode II, the battery’s ultracapacitor energy storage system dominates the voltage of the intermediate circuit when the voltage of the intermediate circuit is between the upper and lower limits of the voltage. In Mode III, the ERP BTS dominates the DC bus when there is a redundant power output to charge the capacitor and generate a DC-link voltage.

### 2.4. Energy Management Using Fuzzy Logic Control

**Figure 3:**
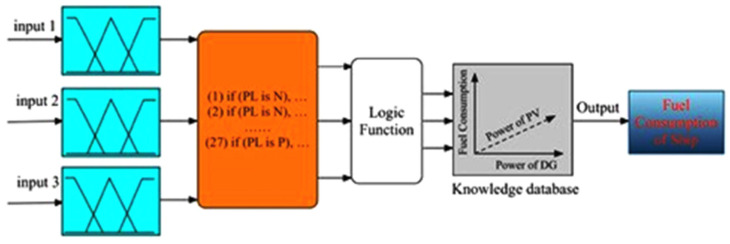
Structure of the simulation model in MATLAB/Simulink.

The FC provides average power in steady state, and the battery has a higher power density than the FC, thus providing a peak power during the transient process. On the basis of a fuzzy logic method, this energy management seeks to extend the performance of the electric vehicle under investigation and the lifetime of the energy source by taking into account two restrictions: FC’s low response and state of charge for battery (SoC). In this paper, the fuzzy logic controller contains three inputs: load energy; SoC for battery; and output, which is the current needed to supply from FC. Matlab/Simulink was used to simulate the entire system. A simulation model based on MATLAB/Simulink (R2017a, MathWorks, Natick, MA, USA) was created to validate the proposed fuzzy-logic-based energy management strategy (Figure 3). FC power, FCV load requirements, and battery SOC are considered input variables. By setting fuzzy rules and logical functions, one can obtain the optimum output power for the FC system and battery. Then, by learning the knowledge base, one can derive the fuel consumption of FCV.

**Figure 4:**
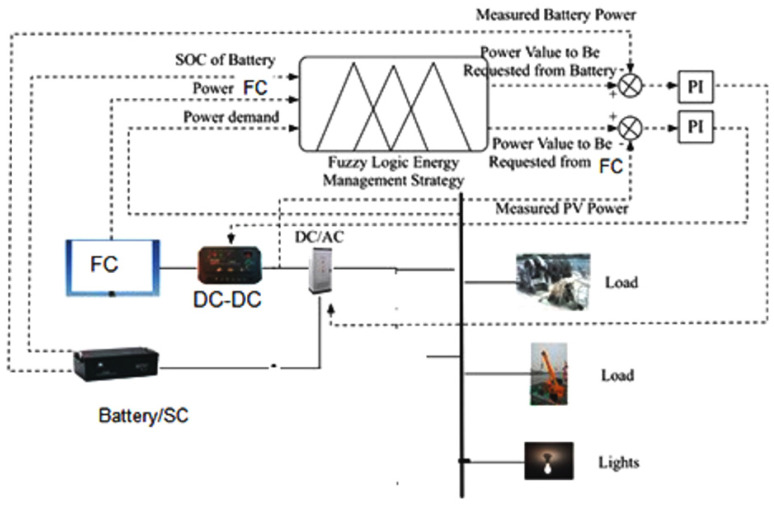
Structure of the energy management strategy based on fuzzy logic.

Under normal navigation conditions, both the propulsion’s load and the power produced by the fuel cell system are undefined and ambiguous due to the uncertainty and time variability of the navigation environment. Therefore, the propulsion’s real-time load requirements, fuel cell energy output power, and battery SOC were used as input variables for fuzzy logical energy management strategies. Input variables and fuzzy rules were used in the calculations according to the logical functions. One can obtain the fuel cell power and the actual reference output of the secondary battery using anti-fuzzy conversion. The PI controller allows one to precisely control the real-time energy output of the battery and fuel cell system, as shown in Figure 4.

**Figure 5:**
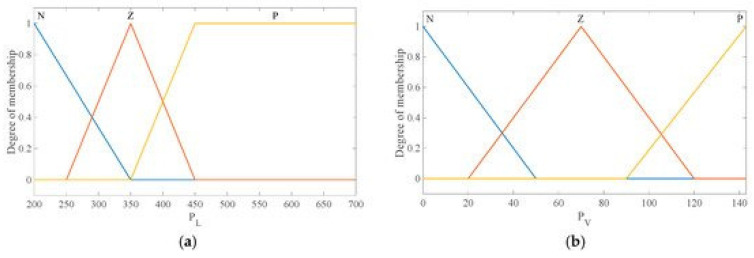

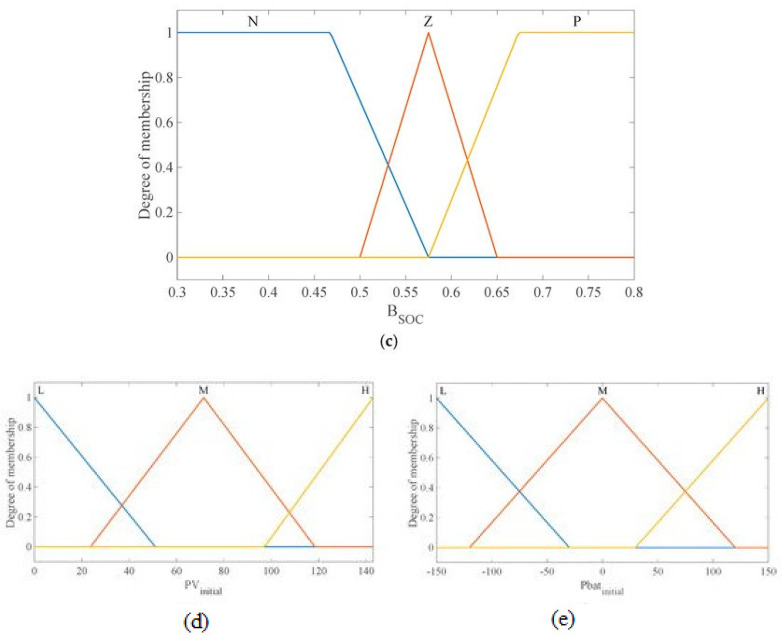
Membership function of the inputs. (**a**) Function of the load power demand. (**b**) Function of the fuel cell energy. (**c**) Function of the battery SOC. (**d**) Function of the initial output power of fuel cell energy. (**e**) Function of the battery initial output power.

For the studied system, the required power load on the electric vehicle was 200–700 kW. According to the hybrid drive system design, FC power was 0 to 143 kW, battery output power was −150 to 150 kW, a negative sign pointed to the state of charge, and a positive sign indicated the state of discharge. The SOC of the battery was set between 0.3 and 0.8 values to restrict fully charged or discharged battery. The fuzzy statistical principles and the corresponding membership function can be obtained from the fuzzy collection of each variable. For input variables, the propulsion’s power requirement (load power demand) *P_L_* was divided into three fuzzy sets: large (P), medium (Z), and small (N). The fuel cell power P_FC_ was divided into three fuzzy sets: large (P), medium (Z), and small (N). The SOC of the battery BSOC was fuzzy in three fuzzy sets: large (P), medium (Z), and small (N). The output variables’ initial fuel cell power P_FCinitial_ and initial battery power *P*_batinitial_ were also fuzzy with three fuzzy sets: high (H), medium (M), and low (L). The input and output membership functions are shown in Figure 5.

To maximize the fuel efficiency of the system, one can set wise fuzzy rules for fuel cell energy system power generation and battery current SOC to meet different real-time load requirements. For example, if the propulsion’s load requirements are high, power from renewable energy systems should be prioritized and maximized to reduce the power supply to diesel generators. If the propulsion does not need much charging, the efficiency of the diesel generator should be taken into account. When the load is low, the fuel consumption rate of the diesel generator can be high, and thus the power from the renewable energy system should be supplied to the grid as much as possible. In this case, the output power of the renewable energy system should be adjusted within a reasonable range. On the basis of the above principles, a total of 27 fuzzy rules were created and written in the fuzzy language as below:

1. If (*P_L_* is N), (P_fc_ is N) and (BSOC is N); then, (P_fcinitial_ is L) and (P_batinitial_ is L);
2. If (*P_L_* is N), (P_fc_ is N) and (BSOC is Z); then, (P_fcinitial_ is L) and (P_batinitial_ is M);
3. If (*P_L_* is N), (P_fc_ is N) and (BSOC is P); then, (P_fcinitial_ is L) and (P_batinitial_ is M);
4. If (*P_L_* is N), (P_fc_ is Z) and (BSOC is N); then, (P_fcinitial_ is M) and (P_batinitial_ is L).

## 3. Results and Discussion

**Figure 6:**
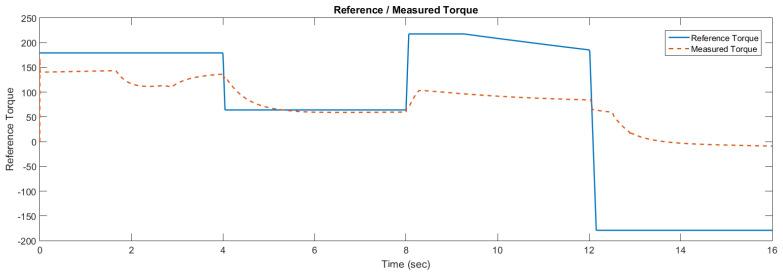
Reference and measured torque in the case of the supercapacitor.

**Figure 7:**
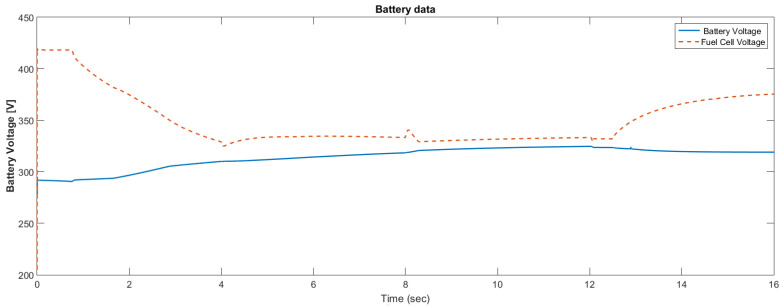
Fuel cell voltage using the supercapacitor.

**Figure 8:**
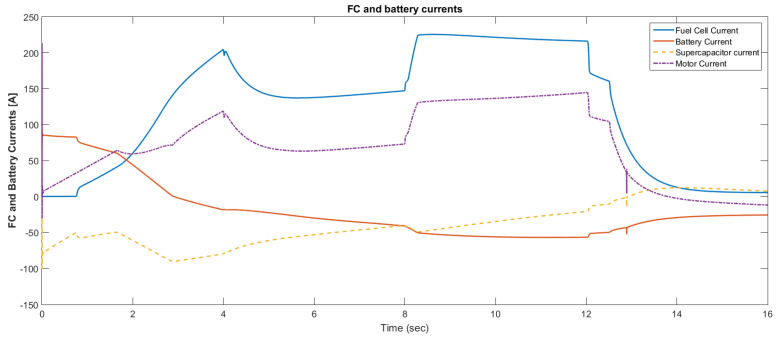
Current after using the supercapacitor.

**Figure 9:**
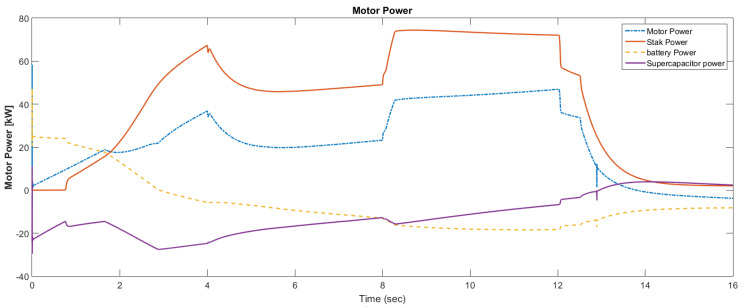
Motor, FC, battery, and supercapacitor powers.

The simulation model was designed using MATLAB/Simulink. The simulation studies were carried out and conducted using MATLAB/Simulink. The main purpose of this study was to investigate the dynamic behavior of the proposed powertrain during traction, braking, and operating modes. These figures show the proposed powertrain dynamic response between traction and braking modes. These operating cycles of the proposed fuel cell vehicle were speed sequences with numerous start and stop processes that represent traffic conditions and a representative driving environment. The measured and reference electromagnetic torques are provided in Figure 6. The control loop outputted the reference electromagnetic torque of the PMSM motor. The reference direct and quadrature (dq) components of the stator current corresponding to the commanded torque were derived from the vector control strategy. The output voltage of the fuel cell and battery terminal voltage are shown in Figure 7. The variation of voltage was due to the variation of load and supercapacitor charging requirements. The currents of load and the supercapacitor were supplied by both the battery and fuel cell. The drawn current from the fuel cell and the supplied current to the motor are shown in Figure 8. The related supplied and delivered powers are shown in Figure 9. The power management scheme (PMS) is essential to achieve certain hybridization and accomplish the main goal of determining the best balance between different energy sources.

**Figure 10:**
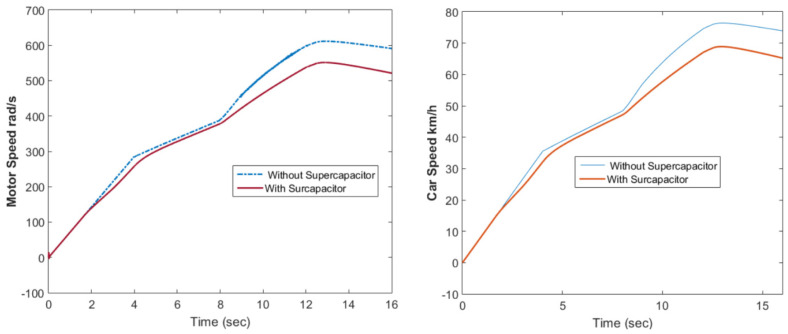
Motor rotational speed and car speed with and without the supercapacitor.

**Figure 11:**
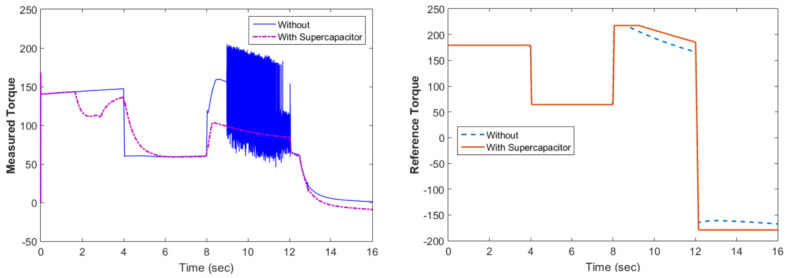
The measured and reference torque with and without the supercapacitor.

**Figure 12:**
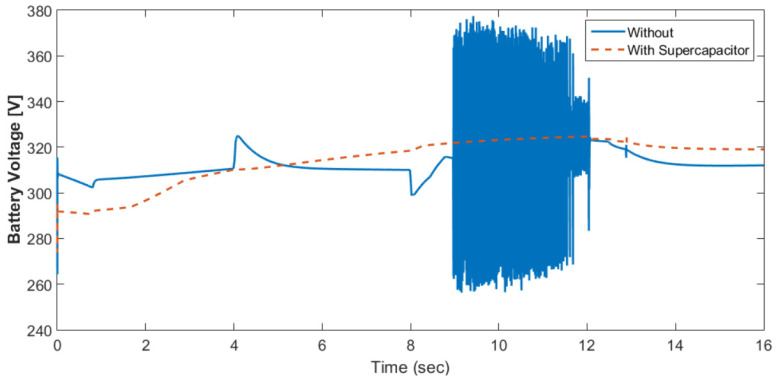
Battery voltage with and without the supercapacitor.

**Figure 13:**
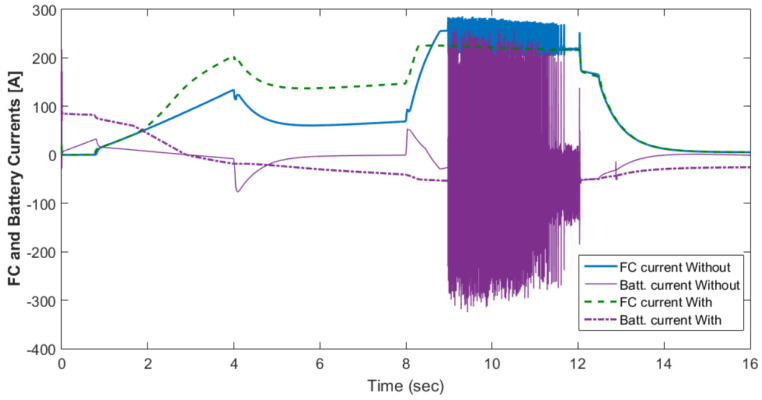
Battery and fuel cell currents before and after using the supercapacitor.

**Figure 14:**
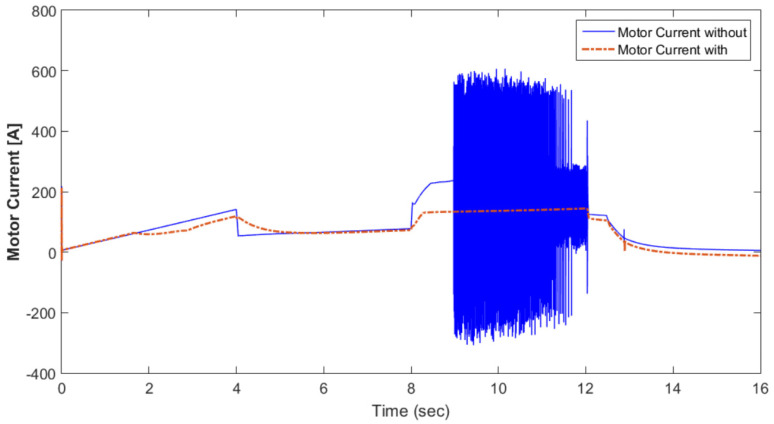
Motor current with and without the supercapacitor.

Figure 10 shows the motor rotational speed and car speed with and without the supercapacitor. The rotational speed was reduced because the supercapacitor was in the charging condition. The measured and reference torques with and without the supercapacitor are shown in Figure 11. In the case without the supercapacitor, there was a torque ripple. This could be eliminated using the supercapacitor. The battery voltage with and without the supercapacitor is shown in Figure 12. As shown in this figure, the voltage ripples were greatly eliminated using the supercapacitor. The battery and fuel cell currents before and after using the supercapacitor are shown in Figure 13. Figure 14 shows the motor current with and without the supercapacitor. The current ripples in Figure 13 and Figure 14 are eliminated using the supercapacitor.

The battery voltage after using the supercapacitor was free of ripples that can damage the battery. The current ripples were mitigated using the supercapacitor. This reduced harmonics in the motor and prevented heating and vibrations on the vehicle. The above results show the performance of the fuel cell electric vehicle with and without using the supercapacitor. For example, motor rotational speed reduced using the supercapacitor. The battery voltage increased using the supercapacitor. The current drawn by the motor was reduced by using the supercapacitor.

### Fuzzy Logic Control Results

**Figure 15:**
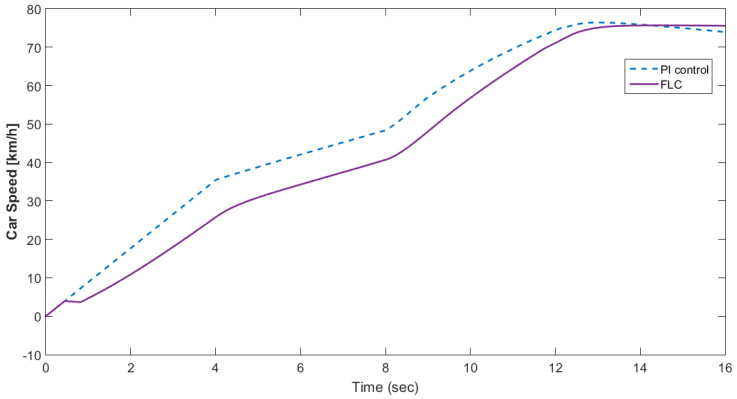
Car speed with PI and fuzzy control.

**Figure 16:**
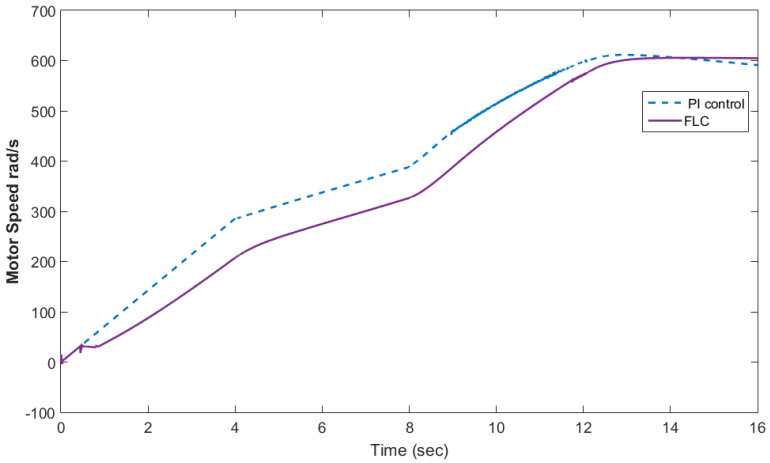
Motor speed with PI and FLC control.

**Figure 17:**
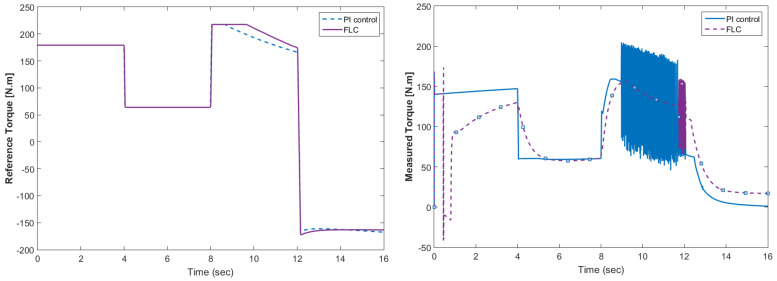
Reference and measured torque in the case of PI and FLC control.

**Figure 18:**
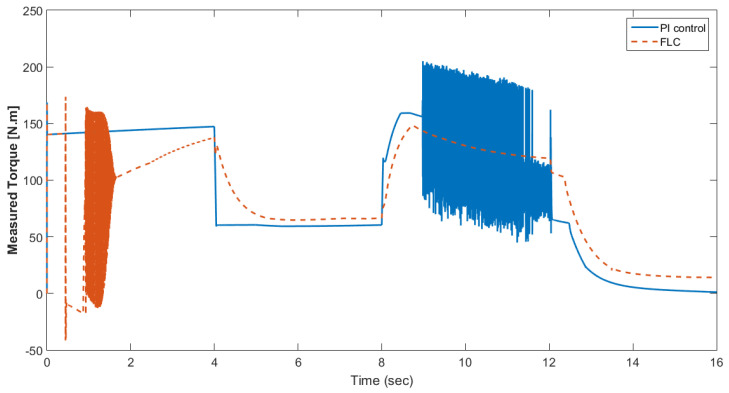
Measured torque with PI and FLC control.

**Figure 19:**
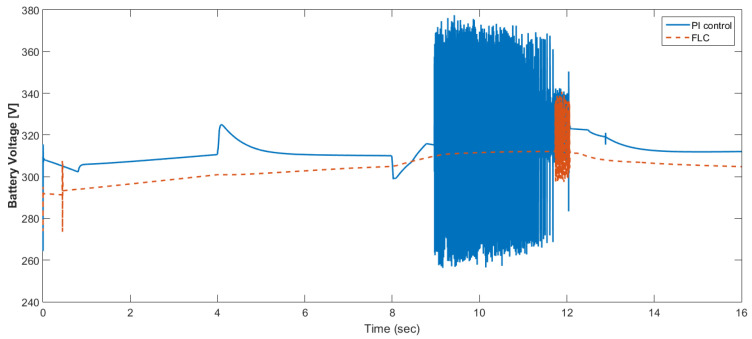
Battery voltage with PI and FLC control.

**Figure 20:**
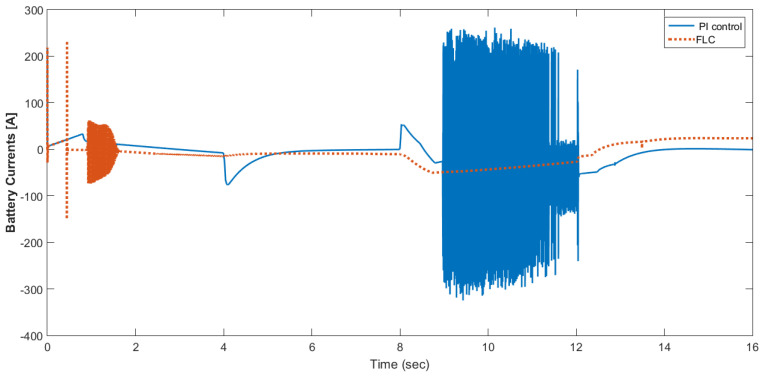
Battery current with PI and FLC control.

**Figure 21:**
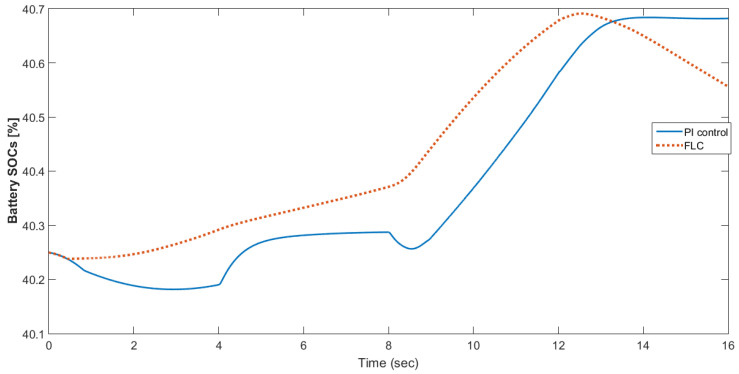
Battery SOC% with PI and fuzzy logic control.

**Figure 22:**
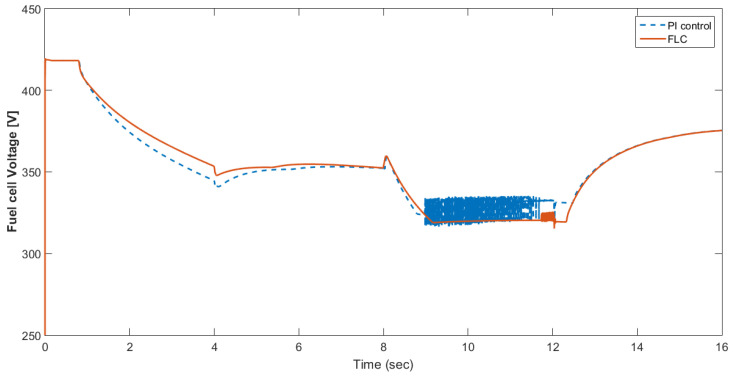
Fuel cell voltage with PI and FLC control.

**Figure 23:**
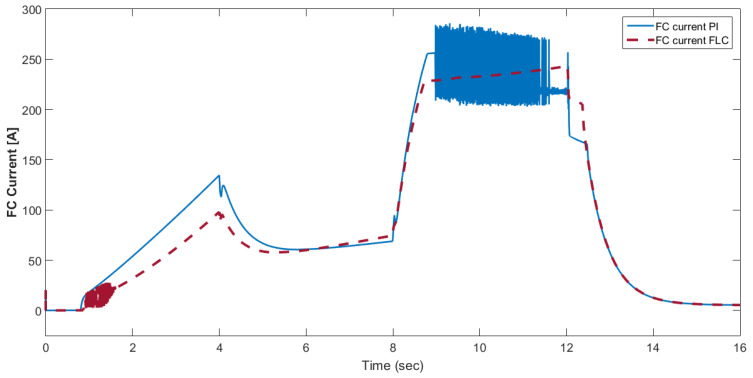
Fuel cell current with PI and FLC control.

**Figure 24:**
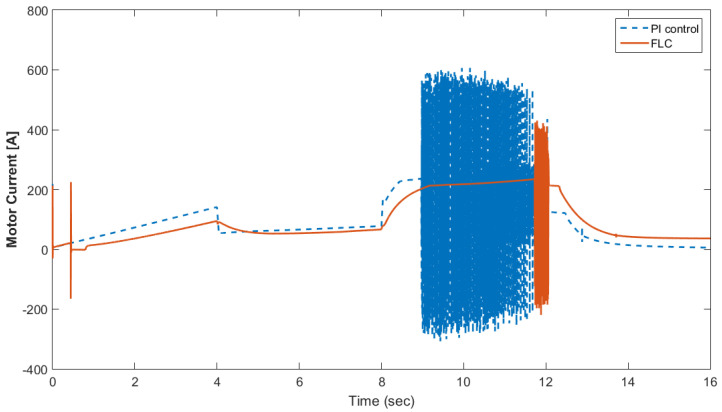
Motor current before and after using FLC.

**Figure 25:**
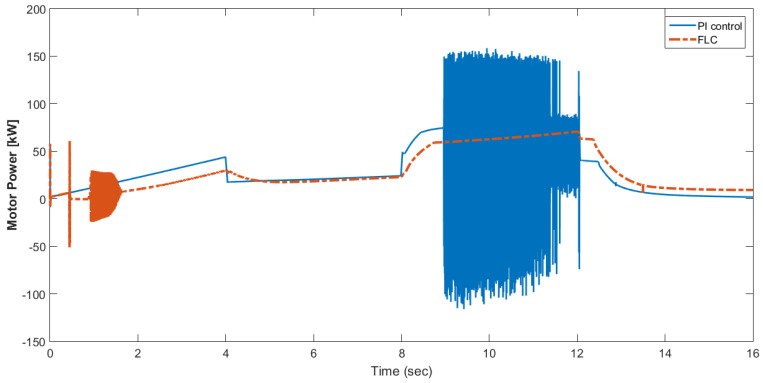
Motor power in kW before and after using fuzzy logic control.

**Figure 26:**
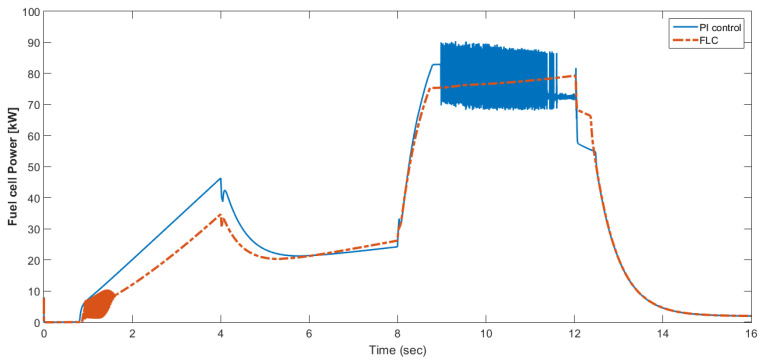
Fuel cell generated power in kW with PI and FLC control.

**Figure 27:**
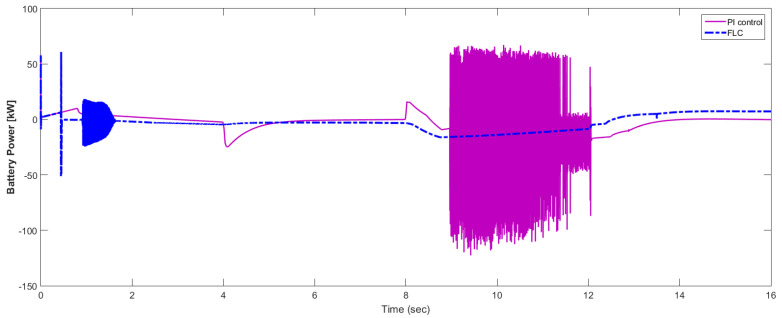
Battery power with PI and FLC control.

Figure 15 shows the car speed with PI and fuzzy control. The speed control loop used a PI regulator to produce the flux and torque references for the vector control. The vector control block computed the three references of motor line currents corresponding to the flux and torque references and then supplied the motor with these currents using a three-phase current regulator. Figure 16 shows the motor speed with PI and FLC control. Figure 17 shows the reference and measured torque in the case of PI and FLC control. Figure 18 shows the measured torque with PI and FLC control. Figure 19 shows the battery voltage with PI and FLC control. Figure 20 shows the battery current with PI and FLC control. The battery SOC% with PI and fuzzy logic control is shown in Figure 21. The fuel cell voltage with PI and FLC control is shown in Figure 22. Figure 23 shows the fuel cell current with PI and FLC control. Figure 24 shows the motor current before and after using FLC. Figure 25 shows the motor power in kW before and after using fuzzy logic control. Figure 26 shows the fuel-cell-generated power in kW with PI and FLC control. Figure 27 shows the battery power with PI and FLC control.

## 4. Conclusions

The measurements confirm the practical feasibility of the proposed EMS by showing that the fuel cell system stabilized the SOC of the battery. For hybrid electric vehicle applications, supercapacitors provide much higher power density than traditional batteries. The design presented seeks to take full advantage of cheaper technology, announcing a supercapacitor energy storage system for fuel-cell-powered hybrid buses. The size of the supercapacitor met the energy storage and requirements of a fuel cell bus. The primary benefit was increased power densities as compared to chemical batteries. The results were illustrated by comparing curves with and without the supercapacitor. The results obtained demonstrate the effectiveness of the proposed power management strategy for performing good power sharing between the two power sources while maintaining the constraints imposed. A simulation model of an FC power-train is presented. The performance of the studied model was investigated by comparing the results of PI and fuzzy logic control. On this basis, the technical parameters of each component and the power structure were also included. The efficiency of the electric vehicle engine and the energy storage function were modeled using standard and conventional PID speed control. The simulation results prove that FLC regulation contributed to the optimization of the energy storage control strategy for FC-EV, and its dynamic and static characteristics were significantly improved.

## Figures and Tables

**Table 1:** FC power-train parameters.

Item	Description
Fuel cell	Nominal operating point Inom = 285 A, Vnom = 300 V
	Number of cells = 400
	Stack nominal efficiency = 57%
	Operation temperature = 95 °C
Super capacitor	Rated capacitance = 15.6 F
	Equivalent Dc series resistance = 150 mOhm
	Rated voltage = 291.6 V
	Series-connected capacitors = 108
	No. of parallel capacitor 1
	Initial voltage = 280 V
	Operating temperature = 25 °C
Li-ion battery	Nominal rated voltage 288 V
	Rating AH capacity 13.9
	Initial state of charge SOC 40.25%
State of charge of the battery	SOCmin–SOCmax: 60–90%
Bidirectional DC–DC converter	A regulated voltage/current at 50 kW output
Inverter system	270 V-DC input, 200 V-AC output, 60 Hz, 150 kVA

**Table 2:** Optimal control scheme for the hybrid AC–DC microgrid.

Dominating Mode	Power Characteristics	Voltage Range	DC-Link Voltage Regulation
Unity (Mode I)	$P_{load}+P_{BTS_{CHG}}+P_{ESS_{CH}}>P_{ESS_{DSC}}+P_{BTS_{DSC}}$	$V_{dcbus}<V_{lower}$	Utility Units
ESS (Mode II)	$P_{ESS_{CH}}<P_{load}+P_{BTS_{CHG}}-P_{BTS_{DSC}}<P_{ESS_{DSC}}$	$V_{lower}<V_{dcbus}<V_{upper}$	ESS Units
ERBTS (Mode III)	$P_{BTS_{DSC}}>P_{load}+P_{BTS_{CHG}}+P_{ESS_{CH}}-P_{ESS_{DSC}}$	$V_{dcbus}>V_{upper}$	ERPBTS Units

## References

[1] Chen Y.-S., Lin S.-M., Hong B.-S. (2013). Experimental Study on a Passive Fuel Cell/Battery Hybrid Power System. Energies.

[2] Long B., Lim S.T., Bai Z.F., Ryu J.H., Chong K.T. (2014). Energy Management and Control of Electric Vehicles, Using Hybrid Power Source in Regenerative Braking Operation. Energies.

[3] Long B., Jeong T.W., Lee J.D., Jung Y.C., Chong K.T. (2015). Energy Management of a Hybrid AC–DC Micro-Grid Based on a Battery Testing System. Energies.

[4] EG&G Technical Services, Inc (2004). Fuel Cell Handbook.

[5] Tang T., Han J., Yao G., Feng Y. Development of a PEM Fuel Cell Boat. Proceedings of the 12th International Power Electronics and Motion Control Conference (EPE-PEMC).

[6] Zemships—Zero Emission Ships. One Hundred Passengers and Zero Emissions: The First Ever Passenger Vessel to Sail Propelled by Fuel Cells. http://ec.europa.eu/environment/life/project/Projects/index.cfm?fuseaction=home.showFile&rep=file&fil=Zemships_Bro-chure_EN.pdf.

[7] Lovers Amsterdam CO2 Zero Canal Cruise. http://www.lovers.nl/co2zero/.

[8] Viking Lady. http://www.vikinglady.no/.

[9] Alkaner S., Zhou P. (2006). A comparative study of lice cycle analysis of molten carbon fuel cells and diesel engines for marine ap-plication. J. Power Sources.

[10] Bensaid S., Specchia S., Federici F., Saracco G., Specchia V. (2009). MCFC-based marine APU: Comparison between conventional ATR and cracking coupled with SR integrated inside the stack pressurized vessel. Int. J. Hydrog. Energy.

[11] Wei J., Fang R. Performance Prediction and Dynamic Simulation of Electric Ship Hybrid Power System. Proceedings of the IEEE Electric Ship Technologies Symposium.

[12] Chao C.-H., Shieh J.-J. (2012). A new control strategy for hybrid fuel cell-battery power systems with improved efficiency. Int. J. Hydrog. Energy.

[13] Gao D., Jin Z., Lu Q. (2008). Energy management strategy based on fuzzy logic for a fuel cell hybrid bus. J. Power Sources.

[14] Wang X., He H., Sun F., Sun X., Tang H. (2013). Comparative Study on Different Energy Management Strategies for Plug-In Hybrid Electric Vehicles. Energies.

[15] Hredzak B., Agelidis V.G., Jang M. (2014). A Model Predictive Control System for a Hybrid Battery-Ultracapacitor Power Source. IEEE Trans. Power Electron..

[16] Camara M.B., Gualous H., Gustin F., Berthon A., Dakyo B. (2010). DC/DC Converter Design for Supercapacitor and Battery Power Management in Hybrid Vehicle Applications—Polynomial Control Strategy. IEEE Trans. Ind. Electron..

[17] Garcia P., Fernandez L.M., Garcia C.A., Jurado F. (2010). Energy Management System of Fuel-Cell-Battery Hybrid Tramway. IEEE Trans. Ind. Electron..

[18] Moreno J., Ortuzar M.E., Dixon J.W. (2006). Energy-management system for a hybrid electric vehicle, using ultracapacitors and neural networks. IEEE Trans. Ind. Electron..

[19] Mazumder S., Jedraszczak P. (2011). Evaluation of a SiC dc/dc converter for plug-in hybrid-electric-vehicle at high inlet-coolant temperature. IET Power Electron..

[20] Nguyen V.-S., Tran V.-L., Choi W., Kim D.-W. (2014). Analysis of the Output Ripple of the DC-DC Boost Charger for Li-Ion Batteries. J. Power Electron..

[21] (2015). Effects of AC Ripple Current on VRLA Battery Life.

[22] Uddin K., Moore A.D., Barai A., Marco J. (2016). The effects of high frequency current ripple on electric vehicle battery performance. Appl. Energy.

[23] Hutchinson T., Burgess S., Herrmann G. (2014). Current hybrid-electric powertrain architectures: Applying empirical design data to life cycle assessment and whole-life cost analysis. Appl. Energy.

[24] Erickson R.W., Maksimovic D. (2007). Fundamentals of Power Electronics.

[25] Mi C., Peng F.Z., Kelly K.J., O’Keefe M., Hassani V. (2008). Topology, design, analysis and thermal management of power electronics for hybrid electric vehicle applications. Int. J. Electr. Hybrid Veh..

[26] Yilmaz M., Krein P.T. (2013). Review of battery charger topologies, charging power levels, and infrastructure for plug-in electric and hybrid vehicles. IEEE Trans. Power Electron..

[27] Broussely M., Biensan P., Bonhomme F., Blanchard P., Herreyre S., Nechev K., Staniewicz R. (2005). Main aging mechanisms in Li ion batteries. J. Power Sources.

[28] Douglas H., Pillay P. Sizing ultracapacitors for hybrid electric vehicles. Proceedings of the 31st Annual Conference of IEEE Industrial Electronics Society, IECON 2005.

[29] Miller J., Smith R. (2003). Ultra-Capacitor Assisted Electric Drives for Transportation.

[30] Ju F., Zhang Q., Deng W., Li J. Review of structures and control of battery-supercapacitor hybrid energy storage system for electric vehicles. Proceedings of the IEEE International Conference on Automation Science and Engineering.

[31] Kerns B., Lindsay T., Williams T., Eberle W. A control algorithm to reduce electric vehicle battery pack RMS currents enabling a minimally sized supercapacitor pack. Proceedings of the IEEE Transportation Electrification Conference and Expo.

[32] Guidi G., Undeland T., Hori Y. Effectiveness of super capacitors as power-assist in pure EV using a sodium-nickel chloride battery as main energy storage. Proceedings of the International Battery, Hybrid and Fuel Cell Electric Vehicle Symposium.

[33] Khaligh A., Li Z. (2010). Battery, Ultracapacitor, Fuel Cell, and Hybrid Energy Storage Systems for Electric, Hybrid Electric, Fuel Cell, and Plug-In Hybrid Electric Vehicles: State of the Art. IEEE Trans. Veh. Technol..

[34] Sayed K. (2019). ZVS Soft-Switched DC-DC Converter based Charger for Low Voltage battery in Hybrid Electric Vehicles. IET Power Electron..

[35] Ibrahim H., Sayed K., Kassem A., Mustafa R. (2019). Power Management Strategy for Battery Electric Vehicles. IET Electr. Syst. Transp..

[36] Sayed K., Abo-Khalil A.G., Alghamdi A.S. (2019). Optimum Resilient Operation and Control DC Microgrid Based Electric Vehicles Charging Station Powered by Renewable Energy Sources. Energies.

[37] Li Q., Chen W., Li Y., Liu S., Huang J. (2012). Energy management strategy for fuel cell/battery/ultracapacitor hybrid vehicle based on fuzzy logic. Int. J. Electr. Power Energy Syst..

[38] Yuan Y., Zhang T., Shen B., Yan X., Long T. (2018). A Fuzzy Logic Energy Management Strategy for a Photovoltaic/Diesel/Battery Hybrid Ship Based on Experimental Database. Energies.

[39] Almutairi A., Sayed K., Albagami N., Abo-Khalil A., Saleeb H. (2021). Multi-Port PWM DC-DC Power Converter for Renewable Energy Applications. Energies.

[40] Sayed K., Kassem A., Saleeb H., Alghamdi A., Abo-Khalil A. (2020). Energy-Saving of Battery Electric Vehicle Powertrain and Efficiency Improvement during Different Standard Driving Cycles. Sustainability.

[41] Kassem R., Sayed K., Kassem A., Mostafa R. (2020). Power optimisation scheme of induction motor using FLC for electric vehicle. IET Electr. Syst. Transp..

[42] Manoharan Y., Hosseini S.E., Butler B., Alzhahrani H., Senior B.T.F., Ashuri T., Krohn J. (2019). Hydrogen Fuel Cell Vehicles; Current Status and Future Prospect. Appl. Sci..

